# Composition, Protein Profile and Rheological Properties of Pseudocereal-Based Protein-Rich Ingredients

**DOI:** 10.3390/foods7050073

**Published:** 2018-05-07

**Authors:** Loreto Alonso-Miravalles, James A. O’Mahony

**Affiliations:** School of Food and Nutritional Sciences, University College Cork, Cork T12 Y337, Ireland; 116221127@umail.ucc.ie

**Keywords:** pseudocereal, cereal, protein-rich ingredients, macronutrient, protein profile, morphology, rheological properties

## Abstract

The objectives of this study were to investigate the nutrient composition, protein profile, morphology, and pasting properties of protein-rich pseudocereal ingredients (quinoa, amaranth, and buckwheat) and compare them to the more common rice and maize flours. Literature concerning protein-rich pseudocereal ingredients is very limited, mainly to protein profiling. The concentrations of macronutrients (i.e., ash, fat, and protein, as well as soluble, insoluble and total dietary fibre) were significantly higher for the protein-rich variants of pseudocereal-based flours than their regular protein content variants and the rice and maize flours. On profiling the protein component using sodium dodecyl sulfate–polyacrylamide gel electrophoresis (SDS-PAGE), all samples showed common bands at ~50 kDa and low molecular weight bands corresponding to the globulin fraction (~50 kDa) and albumin fraction (~10 kDa), respectively; except rice, in which the main protein was glutelin. The morphology of the starch granules was studied using scanning electron microscopy with quinoa and amaranth showing the smallest sized granules, while buckwheat, rice, and maize had the largest starch granules. The pasting properties of the ingredients were generally similar, except for buckwheat and amaranth, which showed the highest and lowest final viscosity, respectively. The results obtained in this study can be used to better understand the functionality and food applications of protein-rich pseudocereal ingredients.

## 1. Introduction

The global protein demand for the 7.3 billion inhabitants of the world is approximately 202 million tonnes annually [1]. The expected continuous growth of the global population to 9.6 billion people by 2050 is creating an ever-greater need to identify and develop sustainable solutions for provision of high-quality food protein [2,3]. Plant-based protein ingredients are becoming more popular due to their contribution to environmental sustainability and to food security challenges, in addition to their cost-effectiveness, compared with animal-based proteins [4]. However, replacing animal-based protein ingredients with plant-origin material is not easy due mainly to important differences in composition and taste/flavour [5]. Moreover, applications of plant proteins are poorly studied and commercially limited due mainly to their techno-functional properties (e.g., poor solubility), anti-nutritional components, off-flavour, and colour [6,7].

Quinoa, amaranth, and buckwheat are non-conventional sources of protein that have been the subject of limited studies in recent years, although their cultivation goes back thousands of years [8,9]. They are gluten-free dicotyledonous grains, referred to as pseudocereals, with somewhat similar composition and nutritional value to cereals, such as rice and maize [10,11]. Quinoa and amaranth are cultivated in South America, and buckwheat, originally from Central Asia, is now also cultivated in Central and Eastern Europe [12]. Their main compositional component is starch [13] which forms semi-crystalline structures referred to as “starch granules”, and depending on the botanical source, these granules vary in size, shape, and amylose:amylopectin ratio [14], which consequently influences the techno-functional properties of the flour ingredients [15,16]. Protein, fibre, fat, minerals, and vitamins are the remaining macro- and micro-nutrients that constitute pseudocereals [9,17]. The protein content of amaranth, buckwheat, and quinoa, has been reported to be 12.0%–18.9% and the concentrations of essential amino acids, particularly, cysteine and methionine, are known to be higher than in some common cereals such as rice and maize [12].

Regarding classification of pseudocereal proteins, the literature in this area is often inconsistent and contradictory [17]. Several authors [18,19] have reported globulins and albumins to be the main proteins in quinoa, amaranth, and buckwheat, in contrast to other cereals, such as rice, where the main proteins are glutelin and prolamins [20]. Amaranth, quinoa, and buckwheat are also good sources of dietary fibre, which has proven effects in promoting desirable physiological outcomes, such as lowering blood cholesterol and increased satiety, due to its resistance to digestion and absorption in the small intestine, followed by complete or partial fermentation in the large intestine [21,22,23]. In addition, pseudocereals are rich in micro-nutrients such as calcium, magnesium, and iron and good sources of vitamin E and riboflavin [24].

**Figure 1:**
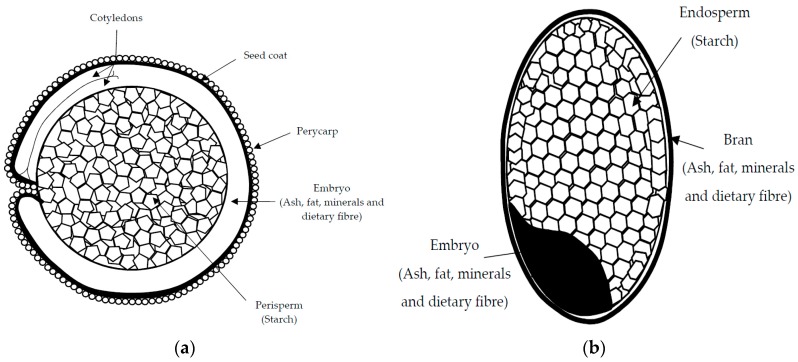

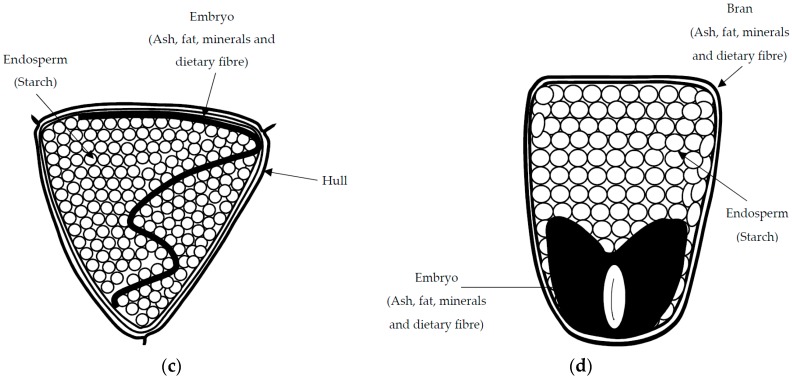
Schematic representation of grain structure of quinoa and amaranth (**a**); rice (**b**); buckwheat (**c**); and maize (**d**).

These macro- and micro-nutrients are located in different parts of the grain (Figure 1). In amaranth and quinoa seeds, the embryo or germ, which is circular in shape, surrounds the starch-rich perisperm, and together with the seed coat, represent the bran fraction, which is relatively rich in fat and protein [25]. In contrast, in buckwheat seeds, starch reserves are stored in the endosperm, as in common cereals, and the embryo, rich in fat and protein, extends through the starchy endosperm [26]. Protein-enriched fractions can be prepared from such pseudocereal grains using two principal approaches—dry or wet fractionation techniques [27]. Dry fractionation employs mechanical forces (milling and air/size classification) and is a more sustainable means of obtaining protein-rich fractions, while wet fractionation techniques use large quantities of water, chemicals (e.g., for pH adjustment), and a final drying step that consumes energy [4,28]. Therefore, protein-rich fractions from pseudocereals can offer unique nutritional and technological properties that have not yet been fully investigated or tested in food applications [29,30,31].

The aim of this work was to determine systematically the nutritional composition, protein profile, and physical properties of several novel protein-enriched ingredients from quinoa, amaranth, and buckwheat and compare them to regular protein content pseudocereal and cereal flours. These protein-rich fractions have great potential as ingredients, not only for their nutritional value (e.g., rich in protein and fibre) but also for their technological functionality (e.g., starch pasting properties). Scientific information on pseudocereal protein-rich fractions is scarce in the literature, thus, the results of this original and novel study can help with our understanding of the potential applications of these plant-based protein-rich ingredients in food formulations.

## 2. Materials and Methods

### 2.1. Cereal and Pseudocereal Flour Ingredients

Ten different regular and protein-rich cereal and pseudocereal flours/ingredients were analysed in this study. Seven of the flours were of pseudocereal origin: quinoa wholegrain flour (QWGF), quinoa dehulled flour (QDF), quinoa protein-rich flour (QPRF), amaranth wholegrain flour (AWGF), amaranth protein-rich flour (APRF), buckwheat dehulled flour (BDF), and buckwheat protein-rich flour (BPRF). Protein enrichment in the protein-rich flours was achieved using a dry milling approach. In brief, the grains were milled using either an impact or a jet mill, with different screen inserts used to produce flour and seed fragments; only buckwheat was milled using a jet mill. All grains, except amaranth, were sourced from commercial suppliers and had been de-hulled prior to milling. After milling, the protein-rich fractions were separated from the milled flours using size-based dry sieve classification. Rice flour (RF), rice protein concentrate (RPC), and maize flour (MF) were included in the study as comparator flour ingredients and were of cereal origin. All of the pseudocereal flours were provided by the Fraunhofer Institut (Munich, Germany) except the QWGF, which was purchased from Ziegler & Co. (Wunsiedel, Germany). The RF and RPC ingredients were purchased from Beneo (Tienen, Belgium) and the MF was purchased from the Quay Co-op (Cork, Ireland).

### 2.2. Chemical Composition

Moisture, ash, fat, and protein contents of samples were determined according to the standard methods of the Association of Analytical Chemists [32]. Moisture was determined by oven drying at 103 °C for 5 h (AOAC 925.10). The ash content was analysed by dry ashing in a muffle furnace at 500 °C for 5 h (AOAC 923.03). Fat determination was carried out following AOAC 922.06, using a Soxtec 2055 (Foss, Ballymount, Co., Dublin, Ireland). Total nitrogen content was determined by the Kjeldahl method (AOAC 930.29) using the following nitrogen-to-protein conversion factors: 6.25 for quinoa, buckwheat, and maize [12,33], 5.85 for amaranth [24], and 5.95 for rice ingredients [12]. Total carbohydrate was calculated by difference (i.e., 100—sum of protein, fat, ash, and moisture). Total starch (AOAC Methods 996.11 and AACC Method 76-13.01), damaged starch as a % of total starch (AACC method 76-31.01 and ICC method No. 164), and soluble (SDF), insoluble (IDF), and total dietary fibre (TDF) (AOAC Method 991.43 and AACC Method 32-07.01) contents were determined using enzyme kits (Megazyme, Bray, Co., Wicklow, Ireland). β-glucan, casein, and high-amylose maize starch were used as controls in dietary fibre analysis (K-TDFC; Megazyme, Wicklow, Ireland).

### 2.3. Electrophoretic Protein Profile Analysis

The protein profile was assessed by sodium dodecyl sulphate-polyacrylamide gel electrophoresis (SDS-PAGE) using precast gels (Mini-PROTEAN TGX, Bio-Rad Laboratories, Hercules, CA, USA) under non-reducing (method **I** and **II**) and reducing conditions (method **III**). The sample loading buffer contained 65.8 mM Tris-HCl (pH 6.8), 26.3% glycerol, 2.1% sodium dodecyl sulfate (SDS) and 0.01% bromophenol blue. The running buffer (10× Tris/Glycine/SDS, Bio-Rad Laboratories, Hercules, CA, USA) had a composition of 25 mM Tris, 192 mM glycine, and 0.1% SDS (*w*/*v*), pH 8.3. The staining solution used was Coomassie Brilliant Blue R-250 (Bio-Rad Laboratories, Hercules, CA, USA). The target final protein concentration was, in all cases, 1 mg/mL, and 10 µL of sample solution loaded into each well of the gel. For the preparation of the samples, three different methods were used. For method **I**, the approach of Abugoch et al. [34] was followed, with slight modifications. Briefly, the powder samples were mixed directly with the sample loading buffer at a concentration of 1 mg/mL, vortexed for 1 min until the powder was fully suspended and mixed over 2 h at 20 °C and at 250 rpm. For methods **II** and **III**, the approach of Amagliani et al. [20] was followed, with the modification that the powders were mixed with the protein extracting buffer overnight, and 1,4-dithiothreitol (DTT; 1%) was used in method **III** as a reducing agent.

### 2.4. Microstructural Analysis

The powders were mounted on aluminium stubs using double-sided adhesive carbon tape, and sputter coated with a 5 nm layer of gold/palladium (Au:Pd = 80:20) using a Quorum Q150R ES Sputter Coating Unit (Quorum Technologies Ltd., Sussex, UK). Subsequently, the samples were loaded into a sample tube and examined using a JSM-5510 scanning electron microscope (JEOL Ltd., Tokyo, Japan), operated at an accelerating voltage of 5 kV.

### 2.5. Pasting Behaviour

Pasting properties were studied using an AR-G2 controlled-stress rheometer equipped with a starch pasting cell (AR-G2; TA Instruments Ltd., Waters LLC, Leatherhead, UK). The internal diameter of the cell was 36.0 mm, the diameter of the rotor was 32.4 mm, and the gap between the two elements at the geometry base was 0.55 mm. A heating and cooling cycle described by Li et al. [35] was applied to 16% (*w*/*w*) suspensions of flours ingredients at a fixed shear rate of 17 rad/s.

### 2.6. Statistical Analysis

All the analyses were conducted in triplicate. The data generated was subjected to one-way analysis of variance (ANOVA) using R i386 version 3.3.1 (R foundation for statistical computing, Vienna, Austria). A Tukey’s paired comparison test was used to determine statistically significant differences (*p* < 0.05) between mean values for different samples, at a 95% confidence level.

## 3. Results and Discussion

### 3.1. Chemical Composition

The dry matter that remains after moisture removal is commonly referred to as total solids [36]. Protein-rich samples had higher total solids (*p* < 0.05) content than their regular flour counterparts. The higher total solids content of these protein-rich ingredients can be an advantage from a microbiological and chemical stability perspective [37]. Ash refers to substances resulting from the incineration of dry matter in a powder sample and is directly related to the mineral content of the sample [38]. The protein-rich ingredients, QPRF, APRF, BPRF, and RPC showed higher ash contents (3.6%, 6.9%, 3.0%, and 3.4%, respectively) than the regular flours QWGF, QDF, AWGF, BDF, RF, and MF (2.3%, 1.8%, 2.4%, 1.5%, 0.8%, and 0.7%, respectively). Protein-rich flours are usually produced using dry fractionation approaches [28], classifying the parts of the grain that are rich in protein (e.g., embryo fraction) which results in a concomitant increase in other components such as minerals [5,25,39]. These pseudocereal protein-rich fractions with higher ash content would be expected to be enriched in selected minerals such as phosphorus, magnesium, and potassium that are located in embryonic tissues [33,40].

The fat content of the protein-rich ingredients QPRF, APRF, and BPRF (12.8%, 16.6%, and 4.8%, respectively) was significantly higher (*p* < 0.05) than the regular flours (Table 1). The higher fat content of the protein-rich ingredients was expected taking into consideration that the dry fractionation process classifies fractions rich in fat along with protein. Arendt and Zannini [40], reported that in quinoa, 49% of the total fat content is located in the embryo. Gamel et al. [41], reported 45% higher fat content in amaranth protein-rich flours, in comparison with a regular flour, and related it with the association of fat with cell wall materials and protein bodies during the protein enrichment process. BPRF showed the lowest value for fat (4.7%) among the protein-rich ingredients. In this study, the low fat content of BDF is most likely due to its relatively low level of protein enrichment (20%) which suggests lower enrichment in the embryo fraction where most of the fat is located. Also, Alvarez-Jubete et al. [26] stated that the fat content in quinoa and amaranth is two to three times higher than in buckwheat and common cereals. The fact that these pseudocereals have high levels of fat reduce the need for adding fat when these protein-rich flours are used as ingredients (e.g., baked products) where fat plays an important role in texture and flavour [41].

The protein-rich flour ingredients, QPRF, APRF, and BPRF, had values for protein of 33.3%, 38.6%, and 20.5%, respectively. The protein contents for pseudocereal flours ranged from 13.1% to 15.7% which are higher than the protein values for RF (8.2%) and MF (6.4%). These values are in accordance with the study of Mota et al. [12], who reported a protein content for pseudocereals significantly higher than in common cereals such as rice and maize. Moreover, a recent review by Navruz et al. [42] reported the nutritional and health benefits of quinoa, such as protein digestibility values similar to casein and higher lysine levels than other grains.

The values for starch in protein-rich samples were lower (21.4–47.3%) than those for the regular flours (50.5–61.6%). The lower values for starch in the protein-rich ingredients were expected as protein-rich ingredients are more enriched in the embryo fraction (rich in proteins), while the perisperm (quinoa and amaranth) or endosperm (buckwheat) where the starch granules are located, are less abundant. The level of starch damage is related to the process and the conditions (e.g., pressure or shear) used to obtain the protein-rich flour ingredients [43]. Such damage changes the granular structure of starch and influences the rheological and functional properties of the starch granules by modulating their water sorption and swelling capacity [43]. QWGF, QDF, QPRF, AWGF, RF, and MF showed similar levels of damaged starch (~7–12% of total starch); while APRF, BDF, and BPRF had the lowest levels of starch damage (~2%) (Table 1). The differences in damaged starch between the samples are usually related to the severity of the extraction process employed [20]. RPC showed the highest damaged starch content (88.3%), which might have arisen from the use of chemicals and aggressive environmental conditions (temperature and pH) in obtaining high protein levels in the final product [4].

**Figure 2:**
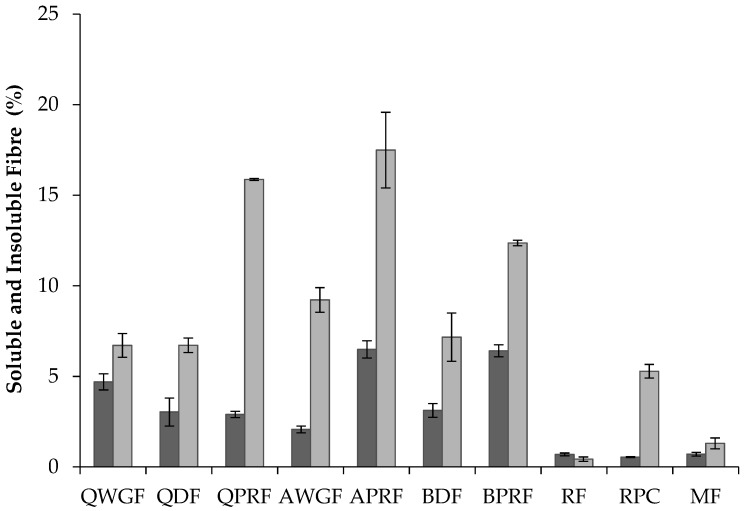
Soluble (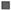) and insoluble (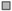) dietary fibre content (% *w*/*w*) of quinoa wholegrain flour (QWGF), quinoa dehulled flour (QDF), quinoa protein-rich flour (QPRF), amaranth wholegrain flour (AWGF), amaranth protein-rich flour (APRF), buckwheat dehulled flour (BDF), buckwheat protein-rich flour (BPRF), rice flour (RF), rice protein concentrate (RPC), and maize flour (MF).

Dietary fibre denotes carbohydrate polymers which are not hydrolysed by the endogenous enzymes in the small intestine of humans [21,22]. Total dietary fibre (TDF) is divided into two categories, based on differences in solubility in water: soluble (SDF) and insoluble (IDF) dietary fibre. Protein-rich cereal ingredients showed significantly higher levels (19–24%) (*p* < 0.05) of TDF than the regular protein containing ingredients (1.1–11.5%) (Table 1 and Figure 2). Among the pseudocereal flours there were no significant differences (*p* < 0.05) in TDF, but they showed higher contents of TDF (*p* < 0.05) in comparison with RF and MF. These results were expected on comparison with literature data: Nascimento et al. [33], reported that pseudocereals can have seven times more fibre than common grains such as rice. The TDF values were similar to those found in other studies for quinoa [33,44,45] where values for TDF of 10.4%, 11.7%, and 12.7%, respectively, were reported, whereas Alvarez-Jubete et al. [26] reported slightly higher values for TDF (14.2%). The value for AWGF is in line with Nascimento et al. [33] who reported a TDF content of 11.3% for amaranth. Other authors, such as Repo-Carrasco et al. [46], reported slightly higher values (ranging from 14% to 16%) for amaranth (*Amaranthus caudatus*) flours. Regarding the soluble and insoluble dietary fibre fractions, the IDF fraction was higher than the SDF fraction in all the ingredients except for RF. This is in accordance with values reported in the literature for quinoa [47,48] and amaranth [19]. However, the IDF content of AWGF was slightly lower than that reported previously by Repo-Carrasco et al. [46] for the varieties Oscar Blanco (12.15%) and Centenario (13.92%). RPC had the lowest values for TDF, SDF, and IDF, which might be explained by the higher protein enrichment levels for this sample, which was in turn, associated with lower levels of other components such as starch, fat, and dietary fibre.

### 3.2. Protein Profile by SDS-PAGE Electrophoresis

**Figure 3:**
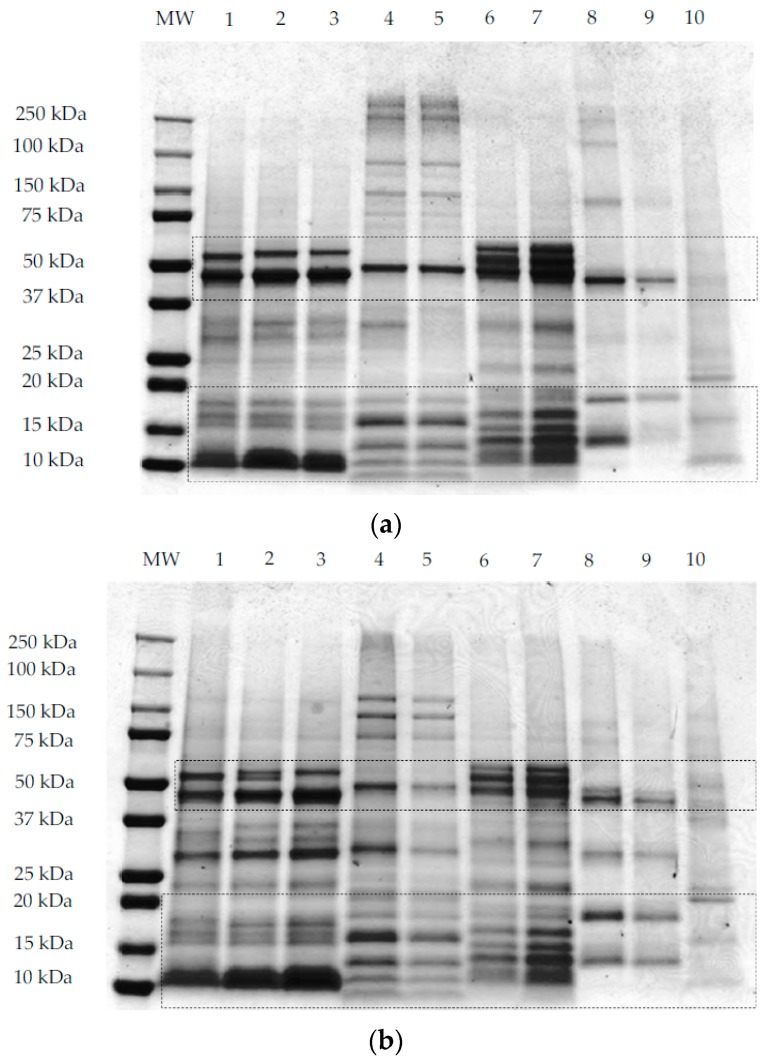

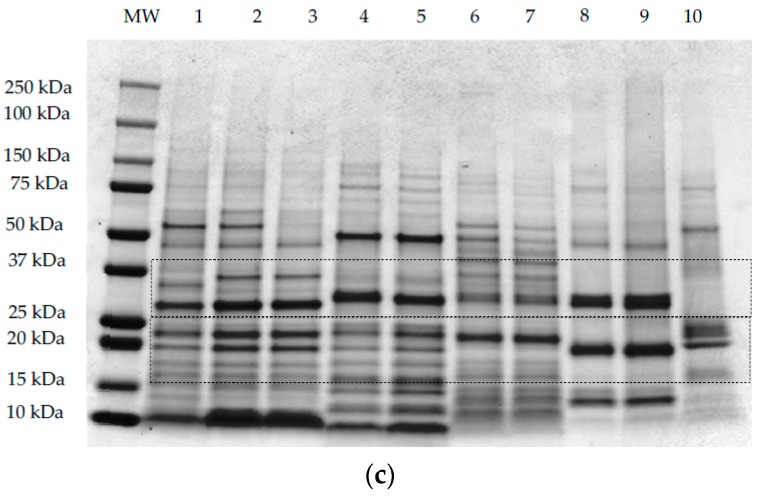
Representative sodium dodecyl sulphate–polyacrylamide gel electrophoresis (SDS-PAGE) patterns of quinoa wholegrain flour (1), quinoa dehulled flour (2), quinoa protein-rich flour (3), buckwheat dehulled flour (4), buckwheat protein-rich flour (5), amaranth wholegrain flour (6), amaranth protein-rich flour (7), rice flour (8), rice protein concentrate (9), and maize flour (10). The first lane of each gel contains the molecular weight marker. Samples were prepared according to methods I, II, and III for gel (**a**–**c**), respectively, as explained in Section 2.3.

SDS-PAGE analyses under non-reducing conditions (Figure 3a,b) and reducing conditions (Figure 3c) were performed using methods **I**, **II,** and **III**, respectively, as outlined in Section 2.3. All samples, except maize, showed common protein bands at ~50 kDa under non-reducing conditions (Figure 3a,b). This band corresponds to the globulin and glutelin fraction in pseudocereals and rice, respectively. For quinoa samples (QWGF, QDF, and QPRF), bands at ~50 kDa (Figure 3a,b) correspond to the 11S globulin fraction, also commonly referred to as chenopodin. Chenopodin consists of ~49 and 57 kDa subunits that are associated into a hexamer by non-covalent interactions [18,49]. When quinoa proteins are treated directly with the sample loading buffer (Figure 3a), two bands with molecular weight (MW) lower and higher than ~50 kDa can be observed. The higher intensity of the lower MW band (Figure 3a,b), suggests that this subunit is predominant in chenopodin protein. When the sample was treated with the protein extracting buffer containing SDS, urea, and thiourea (i.e., under non-reducing conditions; method **II** and Figure 3b), the chenopodin (~50 kDa) did not dissociate into bands of lower MW suggesting that disulphide bonds are the principal linkage between the subunits. In a similar manner to quinoa, the amaranth samples (AWGF and APRF), showed a band at ~50 kDa (Figure 3a,b), which corresponds to the hexameric 11S globulin or amarantin [17]. This major band might also be attributed to another glutelin-type protein which has similar molecular characteristics to those of amaranth 11S globulin [50]. Buckwheat samples, showed a main band at ~50 kDa, which may correspond to the major storage protein of buckwheat, the 13S legume-like globulin, and the minor storage protein, the trimer 8S vicilin-like globulin [51]. Rice samples also showed a major band at ~50 kDa (Figure 3a,b) which corresponds to the glutelin precursor [20].

When the samples were treated with a reducing protein extracting buffer (Figure 3c), the 50 kDa band was disrupted into several bands of lower MW and two of those bands were predominantly around 25–30 kDa and 15–20 kDa, corresponding to the subunits (α- or acidic and β- or basic) that form the globulins for pseudocereals or the glutelins for rice. For quinoa samples treated with the extracting buffer containing DTT as the reducing agent (Figure 3c), it was observed that the disulphide bonds that link the acidic or α- (MW ~28 and 34 kDa) and basic or β- (MW ~17 and 19 kDa) subunits were disrupted, leading to the dissociation of chenopodin into lower MW constituent proteins [28]. The same was observed for amaranth, whereby the acidic or α- (34–36 kDa) and basic or β- (22–24 kDa) subunits of amarantin linked by disulphide bonds are resolved under reducing conditions [17]. Buckwheat 13S legume-like globulin also consists of a small basic subunit (16–29 kDa) linked by a disulphide bond to a large acidic (30–38 kDa) subunit (Figure 3c) [52]. In the case of rice proteins, when the samples are treated with the reducing agent (Figure 3c), the glutelin precursor is disrupted into two main bands with MW ~30 and 20 kDa corresponding to the acidic (α-glutelin) and basic (β-glutelin) subunits that are linked by disulphide bonds. For maize proteins, when the sample was treated with the reducing extracting buffer (Figure 3c), two main protein bands were resolved around 20 kDa that may be related to the main maize protein, zein, a prolamin-like protein that accounts for 60% of the total protein [53].

Bands corresponding to low MW proteins (~10–15 kDa) could be observed in the three gels (Figure 3a–c) for all quinoa, amaranth, and buckwheat samples, which might be related to the albumin fraction, which is abundant in pseudocereals [54,55,56]. For rice samples the band evident at 13 kDa was reported previously as the prolamin fraction [20]. Besides globulin and albumin proteins, amaranth showed high MW proteins (~250 kDa; Figure 3a,b) which were resolved into bands of lower MW under reducing conditions (Figure 3c). Abugoch et al. [34], reported that amaranth glutelin contained an appreciable proportion of aggregated polypeptides of MW greater than 60 kDa. It is possible that the band evident on the gels at ~37 kDa for AWGF sample, and which is not disrupted under reducing conditions, might be the albumin-1 fraction, reported previously to have a MW of 34 kDa [17,55].

### 3.3. Starch Granules: Shape and Size

**Figure 4:**
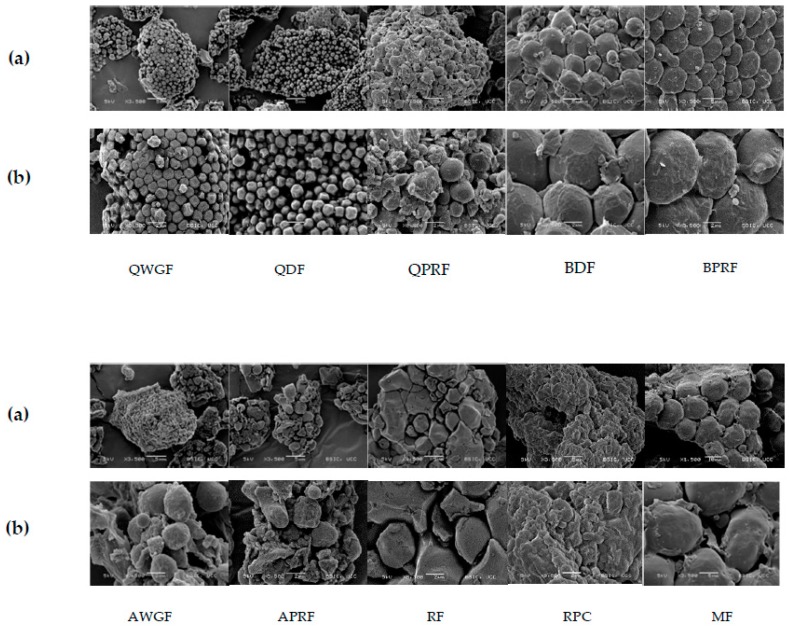
Scanning electron micrographs of quinoa wholegrain flour (QWGF), quinoa dehulled flour (QDF), quinoa protein-rich flour (QPRF), buckwheat dehulled flour (BDF), buckwheat protein-rich flour (BPRF), amaranth wholegrain flour (AWGF), amaranth protein-rich flour (APRF), rice flour (RF), rice protein concentrate (RPC), and maize flour (MF). *Magnification* row (**a**) ×3500; (**b**) ×8500. *Scale bars* row (**a**) 5 µm; (**b**) 2 µm.

Different sizes, shapes, and structures were observed for flour and ingredient powder morphology and ultra-structure using scanning electron microscopy (SEM) analysis (Figure 4). Quinoa samples presented the smallest sized granules (1–1.20 µm) among all samples and had a polygonal shape. The protein-rich flour (QPRF) showed granules covered and linked to other types of substances. This embryo-rich fraction is rich in protein, fibre, and fat which suggests that the starch granules are embedded in a matrix formed by these compounds. Li and Zhu [57] observed that some starch aggregates appeared to be coated with a film-like substance surrounded by a protein matrix. Amaranth samples, AWGF and APRF, showed circular granules with a size of ~2.5–3 µm. Amaranth seed is one of the few sources of small-granule starch, typically 1 to 3 µm in diameter, with a regular granule size [19]. The starch granules in APRF also appeared to be embedded within a matrix as observed for QPRF. Buckwheat starch granules showed the largest size (5 to 7.5 µm) among the pseudocereal samples with a mixture of spherical and polygonal structures. Christa et al. [58], also observed spherical, oval, and polygonal granules with a size distribution from 2 to 6 µm for buckwheat starch. Analysis of the granule structure and matrix positioning showed other components attached which may be protein and fat [59]. The BPRF samples, similar to that observed for QPRF and APRF, also had starch granules embedded in a matrix of other components. Analysis of RF ultrastructure showed starch granules with diameter between 4 and 5 µm, with an angular shape, while maize flour exhibited the largest starch granules (15 µm) with both circular and rod-shapes. These results are in agreement with Nienke et al. [60], who categorized starch granules into different sizes and defined the starch granules for amaranth and quinoa as very small, rice and buckwheat as small, and maize as generally having relatively large granules. The small size of the starch granules of some pseudocereals, such as quinoa, can offer advantages (e.g., altered emulsion stabilisation properties) in respect of incorporation into product formulations [57,61].

### 3.4. Pasting Properties

**Figure 5:**
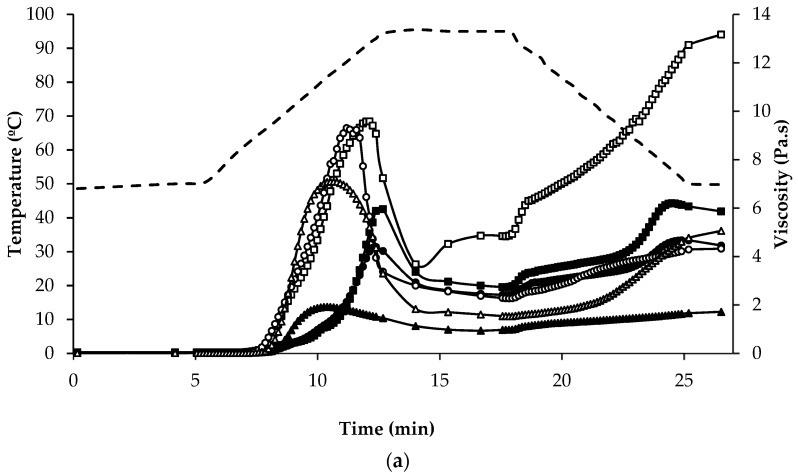

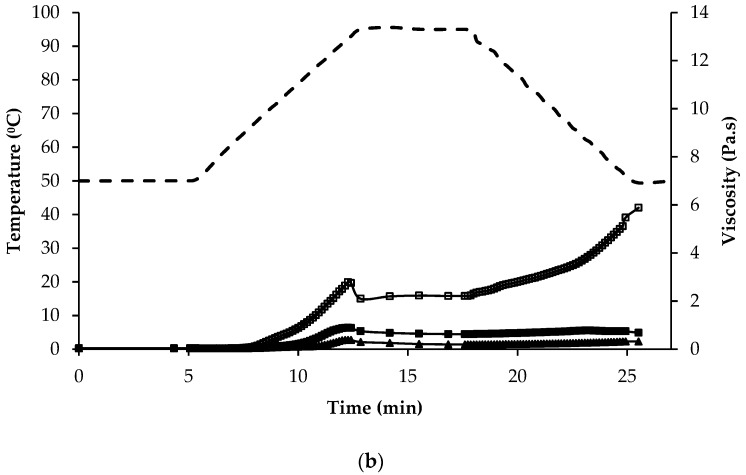
Temperature (dashed line) and viscosity (symbols) at various stages of the pasting regime of (**a**) regular protein containing flours: quinoa wholegrain flour (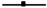), quinoa dehulled flour (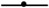), amaranth wholegrain flour (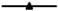), buckwheat dehulled flour (BDF) (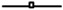), rice flour (RF) (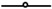), and maize flour (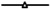); (**b**) of protein enriched flour ingredients: quinoa protein-rich flour (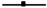), amaranth protein-rich flour (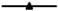), buckwheat protein-rich flour (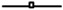).

The mean values for the initial, peak and final viscosity at the end of the holding stage at 95 °C, on completion of cooling to 50 °C, and at the end of the final holding period at 50 °C were recorded during pasting and are presented in Table 2. The shape of the pasting curves differed depending on the type of flour/ingredient (Figure 5a,b). Among the regular protein content flour samples, BDF and RF had the highest viscosity and AWGF the lowest. QWGF and QDF showed slight differences, with QDF having the lowest viscosity; this may be explained by the lower content of starch (50.5%) in QDF than in QWGF (60%). The peak time was very similar for all the flours (~12 min) tested, except MF and BDF which required a shorter time (~10.5 min) to reach peak viscosity, most likely due to the lower extent of absorption and swelling of their starch granules [62]. During the holding period at 95 °C, the material slurries were subjected to high temperature and mechanical shear stress, which further disrupted the starch granules, resulting in the leaching out from starch granules, and alignment, of amylose. It was observed that all the samples displayed a decrease in viscosity, especially so for BDF, RF, and MF, which had the more pronounced decreases in viscosity during the holding period at 95 °C (Figure 5a). The decrease in viscosity during the holding period is often correlated with high peak viscosity: it can be seen how BDF, RF and MF had the highest peak viscosities (Figure 5a). During cooling, re-association between starch molecules, especially amylose chains, will result in the formation of a gel structure and, therefore, viscosity will increase due to retrogradation and reordering of starch molecules. BDF (13.0 Pa∙s) and AWGF (1.72 Pa∙s) showed the highest and lowest final viscosity, respectively, while QWGF, QDF, RF, and MF showed broadly similar final viscosity values (5.83, 4.40, 4.31, and 5.07 Pa∙s, respectively). Regarding the rheological profile of the protein-rich ingredients (Figure 5b), a similar pattern was observed as for the regular protein-content flours in respect of the initial, peak and final viscosities, but with considerably lower viscosity values observed overall. This can be explained by the lower content of starch and the higher content of dietary fibre in the protein-rich samples (Table 2). The water binding capacity of dietary fibre is greatly increased by the presence of high amounts of hydroxyl groups and can be related to a reduction in water availability, which could impact viscosity and pasting properties [63]. Also, the protein-rich flour ingredients are rich in ash, protein, and fat, which have been shown previously to influence the functionality of starch and impact on rheological behaviour of starch dispersions during pasting [59].

Of particular interest, were the high and low viscosity values recorded during pasting for buckwheat and amaranth, respectively. These differences can be related to several factors associated with the starch component of the ingredients, namely the proportion and type of crystalline organization (amylose:amylopectin ratio), size and ultra-structure of the starch granule and extent of starch damage. The amylose content of amaranth and quinoa starch, a component which is related to a stronger and more cohesive gel with higher final paste viscosity [62,64], has been reported to be much lower than that found in buckwheat, rice, or maize [9]. In the case of quinoa starch, the amylose content ranges from 3.5% to 19.6% of total starch, while in amaranth seeds amylose levels have been reported to be lower than 8% [26]. In contrast, the amylose content of buckwheat has been reported to be as high as 57% [58]. Therefore, for buckwheat a higher final viscosity would be expected than for quinoa or amaranth. The starch granule size also influences the pasting temperature, whereby smaller granules have been associated with lower pasting temperatures [60]. BDF had the largest starch granules among the pseudocereal samples analysed in this study (Figure 4) while quinoa and amaranth had the smallest. Yoshimoto et al. [65], reported a higher granule swelling and gelling capacity for buckwheat starches compared with cereal starches. Another factor that can impact the pasting properties is the resistance of starch to digestion by α-amylase during the heating process; Izydorczyk et al. [66] associated the ability of buckwheat to form strong gels with the high resistance of the starch component to digestion by α-amylase. In addition, Lu et al. [67] associated reduced enzyme digestibility of cooked buckwheat groats with retrogradation and formation of resistant starch.

The understanding of the heat-induced rheological behaviour of these protein-rich ingredients is of great importance for the development of tailored nutritional products (e.g., low viscosity in plant-based milk substitutes or high viscosity in yogurt-type products).

## 4. Conclusions

In this study, the nutrient composition, protein profile, and rheological properties of a range of novel protein-rich pseudocereal flour ingredients were studied and compared to regular protein content pseudocereal, maize, and rice flours. The protein-rich flour ingredients had higher levels of ash, fat, and dietary fibre, and lower levels of starch. An integrated proteomic approach was implemented to gain enhanced clarity on the ingredient’s protein profiles, with two strong protein extracting buffers being used for the first time, to allow the complete solubilization and characterization of the proteins in the pseudocereal ingredients. The results showed common bands under non-reducing and reducing conditions that corresponded to the globulin and albumin fractions. The predominance of globulins and albumins in pseudocereals is technologically significant since they are highly soluble in water and dilute salt solutions, which can be an advantage for food formulation purposes, in particular for the production of plant-based beverages. Buckwheat and amaranth had the highest and lowest final viscosity, respectively; while the protein-rich flours had considerably lower viscosity than their regular protein content counterparts. This study provides essential and much-needed new fundamental and applied knowledge on the compositional, structural, and functional properties of protein-rich pseudocereal ingredients to assist in further developing their utilisation in nutritious, functional, and stable food formulations.

## Figures and Tables

**Table 1:** Macronutrient composition of quinoa wholegrain flour (QWGF), quinoa dehulled flour (QDF), quinoa protein-rich flour (QPRF), amaranth wholegrain flour (AWGF), amaranth protein-rich flour (APRF), buckwheat dehulled flour (BDF), buckwheat protein-rich flour (BPRF), rice flour (RF), rice protein concentrate (RPC), and maize flour (MF)*.* Total dietary fibre (TDF). Values are means ± standard deviations of data from triplicate analysis.

	Moisture	Ash	Protein (% *w*/*w*)	Fat	Carbohydrate	Starch	Damaged Starch (% Total Starch)	TDF (% *w*/*w*)
**Quinoa**								
QWGF	9.01 ± 0.10 ^d^	2.30 ± 0.00 ^c^	13.1 ± 0.10 ^b^	6.54 ± 0.07 ^d^	69.0 ± 0.27 ^d^	60.0 ± 2.58 ^d^	10.6 ± 0.47 ^d^	11.4 ± 1.10 ^b^
QDF	8.86 ± 0.25 ^d^	1.80 ± 0.10 ^b^	15.7 ± 0.30 ^b^	5.36 ± 0.61 ^d^	68.3 ± 1.26 ^d^	50.5 ± 1.40 ^bc^	11.7 ± 0.32 ^e^	9.75 ± 1.17 ^b^
QPRF	5.25 ± 0.25 ^a^	3.60 ± 0.19 ^e^	33.3 ± 1.10 ^d^	12.8 ± 0.73 ^e^	45.0 ± 2.27 ^b^	21.4 ± 0.81 ^a^	10.4 ± 0.40 ^d^	18.8 ± 0.23 ^c^
**Amaranth**								
AWGF	8.94 ± 0.05 ^d^	2.40 ± 0.02 ^c^	14.6 ± 0.30 ^b^	6.04 ± 0.10 ^d^	68.1 ± 0.47 ^d^	52.8 ± 1.45 ^c^	12.2 ± 0.35 ^e^	11.3 ± 0.86 ^b^
APRF	7.76 ± 0.12 ^b^	6.86 ± 0.18 ^f^	38.6 ± 1.74 ^e^	16.6 ± 0.08 ^f^	30.2 ± 2.12 ^a^	20.3 ± 0.31 ^a^	2.61 ± 0.01 ^b^	24.0 ± 2.56 ^d^
**Buckwheat**								
BDF	8.75 ± 0.11 ^d^	1.51 ± 0.31 ^b^	14.2 ± 0.06 ^b^	2.77 ± 0.05 ^bc^	72.8 ± 0.53 ^e^	61.6 ± 0.12 ^d^	1.52 ± 0.06 ^a^	10.3 ± 1.72 ^b^
BPRF	6.86 ± 0.17 ^c^	3.05 ± 0.10 ^d^	20.5 ± 0.90 ^c^	4.76 ± 0.15 ^cd^	64.8 ± 1.32 ^c^	47.3 ± 1.20 ^b^	2.22 ± 0.07 ^ab^	19.0 ± 0.48 ^c^
**Rice**								
RF	8.89 ± 0.19 ^d^	0.85 ± 0.05 ^a^	8.22 ± 0.14 ^a^	0.71 ± 0.08 ^a^	81.3 ± 0.46 ^f^	78.5 ± 0.82 ^e^	10.7 ± 0.14 ^f^	1.12 ± 0.20 ^a^
RPC	6.24 ± 0.08 ^a^	3.42 ± 0.24 ^d^	75.0 ± 0.38 ^f^	0.79 ± 0.00 ^a^	14.6 ± 0.7 ^g^	6.50 ± 0.71 ^f^	88.3 ± 0.11 ^g^	5.83 ± 0.41 ^e^
**Maize**								
MF	12.2 ± 0.31 ^e^	0.74 ± 0.04 ^a^	6.42 ± 0.21 ^a^	1.66 ± 0.02 ^ab^	79.0 ± 0.58 ^f^	76.0 ± 2.26 ^e^	7.21 ± 0.25 ^c^	2.00 ± 0.40 ^a^

Values followed by different superscript letters (a–f) in the same column are significantly different (*p* < 0.05).

**Table 2:** Viscosity of quinoa wholegrain flour (QWGF), quinoa dehulled flour (QDF), quinoa protein-rich flour (QPRF), amaranth wholegrain flour (AWGF), amaranth protein-rich flour (APRF), buckwheat dehulled flour (BDF), buckwheat protein-rich flour (BPRF), rice flour (RF), rice protein concentrate (RPC), and maize flour (MF) dispersions at various stages of the pasting regime. Values are means ± standard deviations of data from triplicate analysis.

	Stage of Pasting					
	Initial Viscosity (mPa∙s)	Peak Viscosity (Pa∙s)	Peak Time (min)	End of Holding at 95 °C (Pa∙s)	End of Cooling to 50 °C (Pa∙s)	Final Paste at 50 °C (Pa∙s)
**Quinoa**						
QWGF	18.4 ± 0.76 ^a,b^	6.25 ± 0.18 ^e^	12.5	2.74 ± 0.34 ^e^	6.07 ± 0.30 ^e^	5.83 ± 0.43 ^e^
QDF	24.8 ± 1.05 ^b,c^	4.39 ± 0.16 ^d^	12.5	2.41 ± 0.24 ^c^	4.68 ± 0.19 ^c^	4.40 ± 0.22 ^c^
QPRF	24.1 ± 0.12 ^b,c^	0.91 ± 0.03 ^ab^	12.5	0.63 ± 0.01 ^a^	0.75 ± 0.01 ^a^	0.67 ± 0.01 ^a^
**Amaranth**						
AWGF	29.1 ± 1.32 ^c^	1.92 ± 0.07 ^b,c^	10.3	0.98 ± 0.03 ^b^	1.64 ± 0.06 ^b^	1.72 ± 0.07 ^b^
APRF	26.4 ± 0.84 ^c^	0.29 ± 0.03 ^a^	12.5	0.13 ± 0.01 ^a^	0.19 ± 0.01 ^a^	0.19 ± 0.01 ^a^
**Buckwheat**						
BDF	48.4 ± 2.64 ^e^	9.60 ± 0.46 ^f^	12.1	4.84 ± 0.90 ^f^	12.5 ± 0.47 ^f^	13.0 ± 0.35 ^f^
BPRF	37.5 ± 2.21 ^d^	2.81 ± 0.10 ^c^	12.5	2.23 ± 0.07 ^d^	5.35 ± 0.24 ^d^	5.92 ± 0.24 ^e^
**Rice**						
RF	15.5 ± 0.06 ^a^	9.37 ± 1.43 ^f^	11.2	2.28 ± 0.23 ^c^	4.24 ± 0.15 ^c^	4.31 ± 0.13 ^c^
RPC	17.9 ± 0.13 ^a^	n.d.	n.d.	0.02 ± 0.00 ^g^	0.02 ± 0.00 ^g^	0.02 ± 0.00 ^g^
**Maize**						
MF	16.4 ± 0.02 ^a^	7.11 ± 0.20 ^e^	10.6	1.54 ± 0.15 ^cd^	4.67 ± 0.30 ^cd^	5.07 ± 0.33 ^d^

Values followed by different superscript letters (a–g) in the same column are significantly different (*p* < 0.05). n.d. = not detected.
